# 227. A Randomized, Placebo-controlled Trial to Evaluate the Safety and Efficacy of CD388, a Novel Drug-Fc-Conjugate, for Prevention of Illness due to Influenza A and B in Healthy Unvaccinated Participants

**DOI:** 10.1093/ofid/ofaf695.010

**Published:** 2026-01-11

**Authors:** Nicole Davarpanah, Corrina A Pavetto, Les Tari, Joaquin Sosa, Jennifer A Elder, Frederick Hayden, William J Alexander

**Affiliations:** Cidara Therapeutics, Sandy Spring, Maryland; Cidara Therapeutics, Inc., San Diego, California; Cidara Therapeutics, Sandy Spring, Maryland; Cidara Therapeutics, Sandy Spring, Maryland; PharPoint Research, Inc., Wilmington, North Carolina; Cidara Therapeutics, Inc., San Diego, California

## Abstract

**Background:**

CD388 is a first-in-class drug-Fc-conjugate (DFC) arraying multiple copies of dimeric zanamivir on an engineered human antibody fragment that displays an extended half-life of 6-8 weeks. The median IC_50_s for CD388 against viral neuraminidase of influenza A subtypes and influenza B were ≤ 2.24 nM and ≤ 2.37 nM, respectively, confirming potent antiviral activity. Pharmacokinetic modeling shows that CD388 could protect against symptomatic influenza for up to 6 months.

**Figure:**
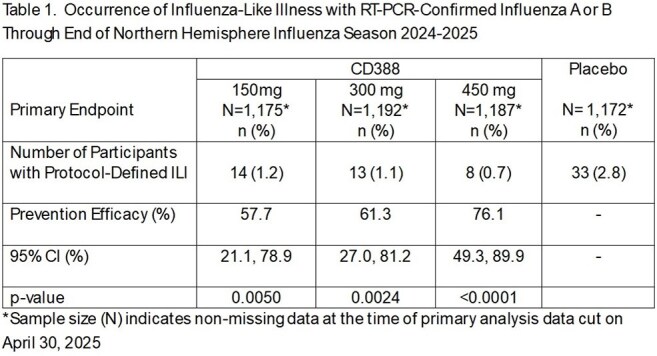

**Figure:**
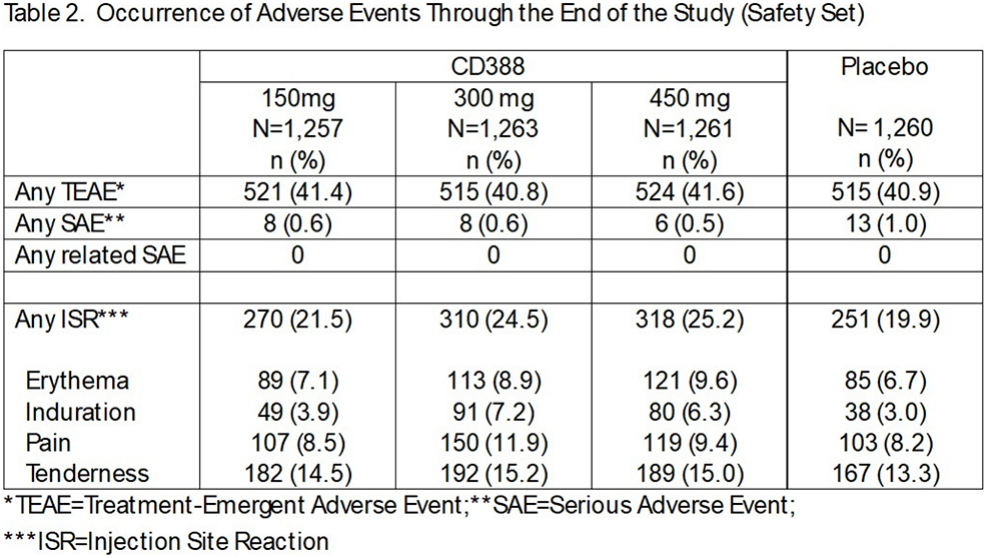

**Methods:**

This double-blind, randomized, placebo-controlled, phase 2 study examined CD388 safety and preventive efficacy against influenza in unvaccinated healthy adults during the 2024-2025 Northern Hemisphere influenza season. Participants (N=5041) were randomized 1:1:1:1 to receive single subcutaneous (SQ) doses of 150 mg, 300 mg, or 450 mg of CD388 or placebo. The primary endpoint was the proportion of participants with proven influenza illness that developed ≥ 7 days up to 24 weeks after study drug dosing, defined by temperature ≥ 38.0^o^C and ≥ 2 respiratory or 1 respiratory and 1 systemic sign/symptom, and influenza virus infection confirmed by reverse transcriptase-polymerase chain reaction from a nasopharyngeal specimen.

**Figure:**
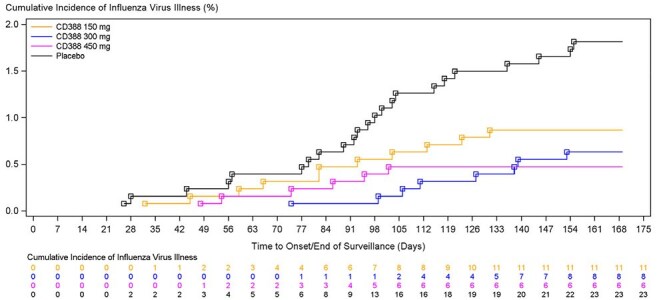

**Results:**

Each CD388 dose provided significant preventive efficacy (PE) versus placebo - 57.7% (CD388 150 mg), 61.3% (CD388 300 mg), and 76.1% (CD388 450 mg) (Table 1). Prevention of influenza illness was greatest in participants receiving CD388 450 mg with minimal loss of efficacy over 24 weeks (Figure) and PK data confirming extended exposure. Local injection site reactions were similar across groups and the incidence of treatment-emergent adverse events was similar to placebo for all CD388 groups (Table 2).

**Conclusion:**

CD388 was well-tolerated and demonstrated high PE for proven influenza illness in unvaccinated participants. Protection exceeded that afforded by seasonal influenza vaccines during the same season. The CD388 dose of 450 mg is targeted for further evaluation. A single dose of CD388 has the potential to provide season-long prevention of illness due to influenza A and B viruses in persons who might not be adequately protected by available influenza vaccines.

**Disclosures:**

All Authors: No reported disclosures

